# Nail in my heart

**DOI:** 10.1186/2036-7902-7-S1-A25

**Published:** 2015-03-09

**Authors:** Siti Nor, Rahimi Abd Razak, S Mohamad Ali, H Ngadiron

**Affiliations:** Emergency and Trauma, Hospital Ampang, Ampang, Malaysia

**Keywords:** Pneumothorax, Emergency Physician, Left Chest, Bedside Ultrasound, Pericardial Window

## Introduction

Stab wound (SW) exploration to assess thoracic or abdominal fascia integrity is a highly invasive procedure which is performed under challenging circumstances in the Emergency Department (ED). Presenting a case report whereby bedside ultrasound was used to detect life threatening thoracic injury secondary to penetrating trauma of the chest.

## Case presentation

23 year old Myamnmar gentleman was brought to the ED, Hospital Ampang. He accidently shot a 5cm nail into his left chest wall while working at a construction site. His airway was patent and vital signs were stable, however complaints of left chest pain with minimal shortness of breath. The nail was firmly cemented to the medial side of his left nipple. Bedside ultrasound was performed by an attending emergency physician. Pneumothorax and hemothorax were able to be ruled out immediately. The most dreadful cardiac injury was promptly ruled out as well as the tip of the nail was found few millimetres just above the pericardium.

Chest xray performed to further confirm the diagnosis. Bedside nail extraction from the chest was performed by surgeon on cal under local anaesthesia. Patient was observed in ED for a day and discharged uneventfully.

## Discussion

Chest injuries results in devastating consequences if prompt treatment is not provided. Clinical assessment is vital however ultrasound has been proven as a useful adjunct in primary survey. Using Extended Focussed Assessment Sonography in Trauma (E-FAST) has allowed prompt diagnosis and intervention.

However in penetrating trauma, use of ultrasound in warranting the need for invasive wound exploration is still a challenge. A positive ultrasound (evidence of pleural effusion, haemothorax or pneumothorax) obviates the need for invasive wound exploration; however, a negative thoracic/FAST (pericardial window) does not rule out the need for local wound exploration. Reasons for this notion is due to several factors such as nature of injury to the pericardial sac, the amount of bleeding into the tract, haematoma within the wound tract and the skill of the ultrasound operator.

## Lesson learnt

A bedside ultrasonography, performed by an emergency physician has the potential to improve patient care by facilitating a more timely diagnosis and expedite intervention. However use of ultrasound in warranting the need for invasive wound exploration in penetrating injury is still a challenge.

## Informed consent

The study was conducted in accordance with the ethical standards dictated by applicable law. Informed consent was obtained from each owner to enrolment in the study and to the inclusion in this article of information that could potentially lead to their identification.

